# Causal association between colorectal cancer and Alzheimer’s disease: a bidirectional two-sample mendelian randomization study

**DOI:** 10.3389/fgene.2023.1180905

**Published:** 2024-01-05

**Authors:** Chunsheng Yuan, Saisai Liu, Kezhen Yang, Feiyu Xie, Yinan Li, Yantong Guo, Wenjun Zhao, Jincheng Zhang, Zhiqiang Cheng

**Affiliations:** ^1^ Graduate School, Beijing University of Chinese Medicine, Beijing, China; ^2^ Department of Integrative Oncology, China-Japan Friendship Hospital, Beijing, China; ^3^ Department of Dermatology, China-Japan Friendship Hospital, Beijing, China; ^4^ School of Acupuncture-Moxibustion and Tuina, Beijing University of Chinese Medicine, Beijing, China; ^5^ Oncology Department, Wangjing Hospital of China Academy of Chinese Medical Sciences, Beijing, China; ^6^ Oncology Department, Beijing Hospital of Traditional Chinese Medicine, Capital Medicine University, Beijing, China

**Keywords:** colorectal cancer, Alzheimer’s disease, two-sample Mendelian randomization, genome-wide association study, causal effect

## Abstract

**Background:** Colorectal cancer and Alzheimer’s disease are both common life-threatening diseases in the elderly population. Some studies suggest a possible inverse relationship between colorectal cancer and Alzheimer’s disease, but real-world research is subject to many biases. We hope to clarify the causal relationship between the two through a bidirectional two-sample Mendelian randomization study.

**Methods:** In our study, we used genetic summary data from large-scale genome-wide association studies to investigate the relationship between colorectal cancer and Alzheimer’s disease. Our primary analysis employed the inverse-variance weighted method and we also used complementary techniques, including MR-Egger, weighted median estimator, and Maximum likelihood. We applied simex adjustment to the MR-Egger results. We also utilized the MRlap package to detect potential sample overlap and its impact on the bias of the results. In addition, we performed several sensitivity and heterogeneity analyses, to ensure the reliability of our results.

**Results:** The combined effect size results of the inverse-variance weighted method indicate that colorectal cancer may decrease the incidence of Alzheimer’s disease, with an odds ratio (OR) of 0.846 (95% CI: 0.762–0.929). Similar results were observed using other methods such as MR-Egger, weighted median estimator, and Maximum likelihood. On the other hand, Alzheimer’s disease may slightly increase the incidence of colorectal cancer, with an OR of 1.014 (95% CI: 1.001–1.027). However, the results of one subgroup were not significant, and the results from MRlap indicated that sample overlap introduced bias into the results. Therefore, the results of the reverse validation are not reliable. The F-statistic for all SNPs was greater than 20. Four SNPs related to the outcome were excluded using Phenoscanner website but the adjustment did not affect the overall direction of the results. The results of these statistics were further validated by MR-PRESSO, funnel plots, leave-one-out analyses, Cochran’s Q, demonstrating the reliability of the findings.

**Conclusion:** According to the findings of this Mendelian randomization study, there appears to be a causal association between colorectal cancer and Alzheimer’s disease. These results could have important implications for clinical practice in terms of how colorectal cancer and Alzheimer’s disease are treated. To better understand the relationship between these two diseases, more research and screening are needed in clinical settings.

## 1 Introduction

Cancer is a major cause of mortality worldwide, presenting a significant obstacle to increasing life expectancy (Bray et al., 2021). Colorectal cancer (CRC) is the third most commonly diagnosed cancer and the second leading cause of cancer-related deaths. In 2020, there were over 1.9 million new cases of CRC and 935,000 deaths globally, comprising approximately 10% of all cancer cases and deaths. It is worth noting that the incidence of CRC varies by a factor of approximately 9 between different regions of the world, with transitioned countries having an incidence rate that is approximately 4 times higher than that of transitioning countries. Among these regions, Europe has the highest incidence rate (Sung et al., 2021). Globally, approximately 50 million people, mostly elderly, are affected by dementia, with an estimated increase to 100–130 million patients between 2040 and 2050 (Taudorf et al., 2021). Alzheimer’s disease (AD) is a degenerative neurological disease that leads to neuronal and synaptic loss, brain atrophy, and ultimately death. It is prevalent among older adults and is the primary cause of dementia, which is a leading cause of mortality in this population. There appears to be no obvious connection between cancer and AD, both of which are age-related conditions. However, according to some research reports, there may be a close or even inverse relationship between the incidence and pathogenesis of cancer and central nervous system diseases, particularly AD (Catalá-López et al., 2014; Jiang et al., 2013). For example, Ferrán *et al.* found a sustained decrease in the overall co-occurrence of cancer in patients with neurodegenerative diseases and AD (Catalá-López et al., 2014). Similarly, a meta-analysis revealed a weak inverse correlation between AD and cancer that cannot be explained by confounding factors, diagnostic bias, or bias in competitive risks. The random-effects meta-analysis indicated that the pooled fixed-effect hazard ratio for AD in CRC survivors was 0.88 (95% CI, 0.80–0.97) compared to individuals without a history of cancer (Ospina-Romero et al., 2020). There are also many studies in the field of basic experiments. For instance, Park *et al.* discovered that inflammatory response is an important mechanism underlying these two phenotypically opposite diseases (Park et al., 2015). Several proteins that suppress tau and amyloid-β deposits, regulate cell cycle (Ma et al., 2012; Li et al., 2013), and undergo common epigenetic modifications (Tremolizzo et al., 2006), as well as age-related metabolic dysfunction (Driver, 2014), are all involved in the pathogenesis of cancer and neurodegeneration. In reality, a portion of patients with AD may experience a decrease in CRC diagnosis rates due to memory loss, cognitive decline, and other factors that prevent them from seeking timely medical examinations such as colonoscopy (Lv et al., 2022). Furthermore, patients diagnosed with cancer often experience emotional stress, invasive surgical treatments, cytotoxic chemotherapy, and persistent pain, which may lead to reduced cognitive function and an increased risk of AD (Aboalela et al., 2015; Hermelink et al., 2015; Whitlock et al., 2017; Ahles and Hurria, 2018). These findings are contrary to many epidemiological and basic experimental research results. In addition, the association between CRC and AD remains unclear due to the bias of reverse causation and confounding factors in traditional observational studies.

Mendelian randomization (MR) employs single nucleotide polymorphisms (SNPs) as instrumental variables (IVs) to establish causality between an exposure and an outcome. Since offspring inherit their alleles from their parents according to Mendelian principles, MR studies resemble randomized controlled trials that occur naturally within populations. The genetic effects of genotypes are relatively stable and less influenced by environmental factors, and all genetic variations occur before disease onset, making it possible to overcome confounding factors and reverse causation (Emdin et al., 2017; Davies et al., 2018). In order to examine the causal relationship between CRC and AD, we performed bidirectional two-sample MR analyses in this study.

## 2 Materials and methods

### 2.1 Study design

To explore the potential relationship between CRC and AD, we utilized summary statistics data from genome-wide association studies (GWASs) and performed a bidirectional two-sample Mendelian randomization study. To conduct a reliable MR study, three core assumptions, namely, relevance, independence, and exclusion restriction, need to be simultaneously satisfied. Further details regarding the MR design can be found here (Davies et al., 2018; Sanderson et al., 2022). Since we are using aggregated data, which is of a de-identified nature and does not constitute human subject research, ethical approval is not required. However, it is important to note that all primary studies included in our analysis obtained ethical approval and obtained informed consent from participants.

### 2.2 Study samples

We utilized three GWAS datasets for MR analysis. In order to avoid population stratification, the analysis only incorporated genetic variants derived from European ancestry. The GWAS dataset related to colorectal cancer is sourced from the United Kingdom Biobank, as reported in the study by Burrows et al., in 2021, which includes 5,657 colorectal cancer patients and 372,016 healthy controls. The GWAS datasets associated with AD are sourced from two databases: The Medical Research Council Integrative Epidemiology Unit (MRC IEU) and the European Bioinformatics Institute (EBI) databases. The study by Ben Elsworth et al., in 2018 includes 19,255 cases and 380,538 controls and the study conducted by Jeremy et al., in 2021 includes 53,042 cases and 355,900 controls (Schwartzentruber et al., 2021).

### 2.3 Instruments selection

Firstly, SNPs (*p* < 5 × 10^−6^) closely associated with colorectal cancer (or Alzheimer’s disease in the reverse validation) were selected from the GWAS database. Typically, SNPs with *p* < 5 × 10^−8^ are considered genome-wide significant, however, selecting SNPs below this threshold yields a limited number of effective SNPs, resulting in reduced statistical power. Therefore, a lower threshold was appropriate. Secondly, the clump step was performed using the TwoSampleMR package in R 4.2.2 software to exclude SNPs in linkage disequilibrium (LD) (Hemani et al., 2018). The specific parameters were set as *R* ^2^ = 0.001 and kb = 10,000, which removed SNPs with *R* ^2^ > 0.001 within a 10-MB range of the most significant SNP. For missing SNPs in the outcome dataset, those with strong LD (*R* ^2^ > 0.8) were used as proxies, and SNPs without alternative sites were removed. Data were then extracted and organized from both datasets to match the exposure and outcome effect values with the same effect allele. To eliminate weak instrumental variables, we introduce the F-statistic (F = beta^2^/se^2^, where beta represents the effect size of the allele and se represents the standard error) to calculate the power of each SNP. We exclude SNPs with an F-statistic less than 10 and compute the average of all F-statistics to represent the overall F-statistic of the IVs (Xie et al., 2023). Then, we remove SNPs that are associated with AD (or CRC in reverse validation) and any phenotypes that might lead to AD using the PhenoScanner website (http://www.phenoscanner.medschl.cam.ac.uk/). Following that, we utilize the MR-Egger regression model’s intercept test and the MRPRESSO method to assess genetic pleiotropy and remove outliers (Verbanck et al., 2018).

### 2.4 Statistical analyses

Four methods were used including inverse-variance weighted (IVW) (Yavorska and Burgess, 2017), MR-Egger (Bowden et al., 2015), weighted median estimator (WM) (Bowden et al., 2016a), and Maximum likelihood (ML) (Yavorska and Burgess, 2017), to estimate the causal association between CRC and AD. The odds ratio (OR) of the outcome variable per 1 log-odds increase in the exposure variable was used to represent the results. The scatter plot was generated using the TwoSampleMR package (Hemani et al., 2018), which includes the most commonly used five methods, including simple mode and weighted mode, to improve the accuracy of causal association assessment. The IVW method requires the regression line to pass through the origin and assumes that all IVs included in the model are valid. Therefore, if there are no pleiotropic IVs in the regression model, IVW can provide an unbiased and efficient estimate of the causal association. The MR-Egger method assumes that the pleiotropy of IVs is unrelated to their effects on exposure and does not require the regression line to pass through the origin. Thus, the intercept term represents the average estimate of genetic pleiotropy, and the slope of the regression line represents the estimate of the true causal association after correcting for genetic pleiotropy (Bowden et al., 2015). When more than 50% of the IVs are valid, the WM method can provide a more accurate estimate of the causal effect, with efficiency approaching that of the IVW method (Wang and Shen, 2020). The Maximum likelihood method is not fundamentally different from the IVW method, but it fully considers uncertainty in genetic associations with both the exposure and outcome, which is ignored in the simple weighting of the IVW method (Yavorska and Burgess, 2017). Additionally, both IVW and MR-Egger methods require the NO Measurement Error (NOME) assumption, and violation of this assumption can result in weak instrument bias. The IVW method can be tested using the F-statistic, while the MR-Egger method can be tested using the *I* ^*2*^_*GX*_ statistic. When *I* ^*2*^_*GX*_ is less than 90%, the simex approach (available in the TwoSampleMR package) should be used for adjustment (Bowden et al., 2016b; Hemani et al., 2018).

Finally, conducting sensitivity analysis to validate the reliability of the statistical results. MRlap employs cross-trait LD score regression (LDSC) to approximate overlap, enabling the evaluation and correction of biases introduced by sample overlap in Mendelian Randomization analyses. Cochran’s Q test and Funnel plots were applied to assess the heterogeneity of SNPs. Using the “leave-one-out” method to assess the magnitude of the causal association effect influenced by individual SNPs.

Analyses were done using the statistical software R (version 4.2.2) with packages TwoSampleMR (version 0.5.6) (Hemani et al., 2018), MRPRESSO (version 1.0), simex (version 1.8), MRlap (version 0.0.3). A significance level of *p* < 0.05 (two-tailed) was considered statistically significant.

## 3 Results

### 3.1 CRC and AD related SNPs

In the study examining the impact of colorectal cancer (CRC) on Alzheimer’s disease (AD) and AD (val), we initially extracted SNPs and identified rs12488768 as correlated with neuroticism score and rs143058554 as correlated with schizophrenia using PhenoScanner website. Since both of these SNPs can promote the onset of Alzheimer’s disease (Low et al., 2013; Ribe et al., 2015), they were subsequently excluded, resulting in the identification of 26 and 28 SNPs, respectively, that were associated with CRC (Table 1). Similarly, in the study examining the impact of AD and AD (val) on CRC, we initially extracted SNPs and identified rs35511257 as correlated with malignant neoplasm of anus and anal canal, and rs2760980 as correlated with inflammatory bowel disease using PhenoScanner website. These two SNPs can promote the onset of colorectal cancer (Shah and Itzkowitz, 2022), and were therefore excluded, resulting in the identification of 19 and 47 SNPs, respectively, that were associated with AD (Table 1). The detailed information of SNPs can be found in Supplementary Tables S1–S4. The intercept of the MR-Egger regression was zero, indicating the absence of genetic pleiotropy between SNPs and both CRC and AD (*p*-values >0.05). Results from the MRPRESSO test similarly showed no evidence of pleiotropic bias (*p*-values >0.05) or outliers (Table 2). All SNPs have an F-statistic greater than 20, indicating that the results are not influenced by weak instrument bias (Table 1). However, since *I* ^*2*^_*GX*_ in the study examining the impact of CRC on AD and AD (val) was less than 90%, the MR-Egger results needed to be adjusted using the simex approach (Table 1) (Bowden et al., 2016b). Nevertheless, the removal of SNPs and the adjustment of MR-Egger results with the simex approach did not significantly affect the causal associations between the two diseases.

**TABLE 1:** Description of samples.

Exposure	CRC	CRC	AD	AD (val)
Outcome	AD	AD (val)	CRC	CRC
SNP	26	28	19	47
F	31.9	31.5	97.4	72.4
*I* ^*2*^_*GX*_	62.6%	72.5%	98.3%	97.2%

Abbreviations: CRC, colorectal cancer; AD, Alzheimer’s disease; val, validation; SNP, single nucleotide polymorphisms.

**TABLE 2:** Directional pleiotropy of MR-Egger test and MR-PRESSO global test.

Exposure	Outcome	Method	Intercept	SE	*p*-value
CRC	AD	MR-Egger	−0.00023	0.00042	0.59
CRC	AD	MR-PRESSO			0.70
CRC	AD (val)	MR-Egger	−0.00013	0.00055	0.81
CRC	AD (val)	MR-PRESSO			0.54
AD	CRC	MR-Egger	0.00012	0.00014	0.43
AD	CRC	MR-PRESSO			0.40
AD (val)	CRC	MR-Egger	−0.000051	0.000084	0.55
AD (val)	CRC	MR-PRESSO			0.10

Abbreviations: CRC, colorectal cancer; AD, Alzheimer’s disease; val, validation; MR-PRESSO, Mendelian randomization pleiotropy RESidual Sum and Outlier; MR-Egger, Mendelian randomization egger.

### 3.2 Estimation of causal relationship between CRC and AD

#### 3.2.1 Causal effect of CRC on AD

**FIGURE 1:**
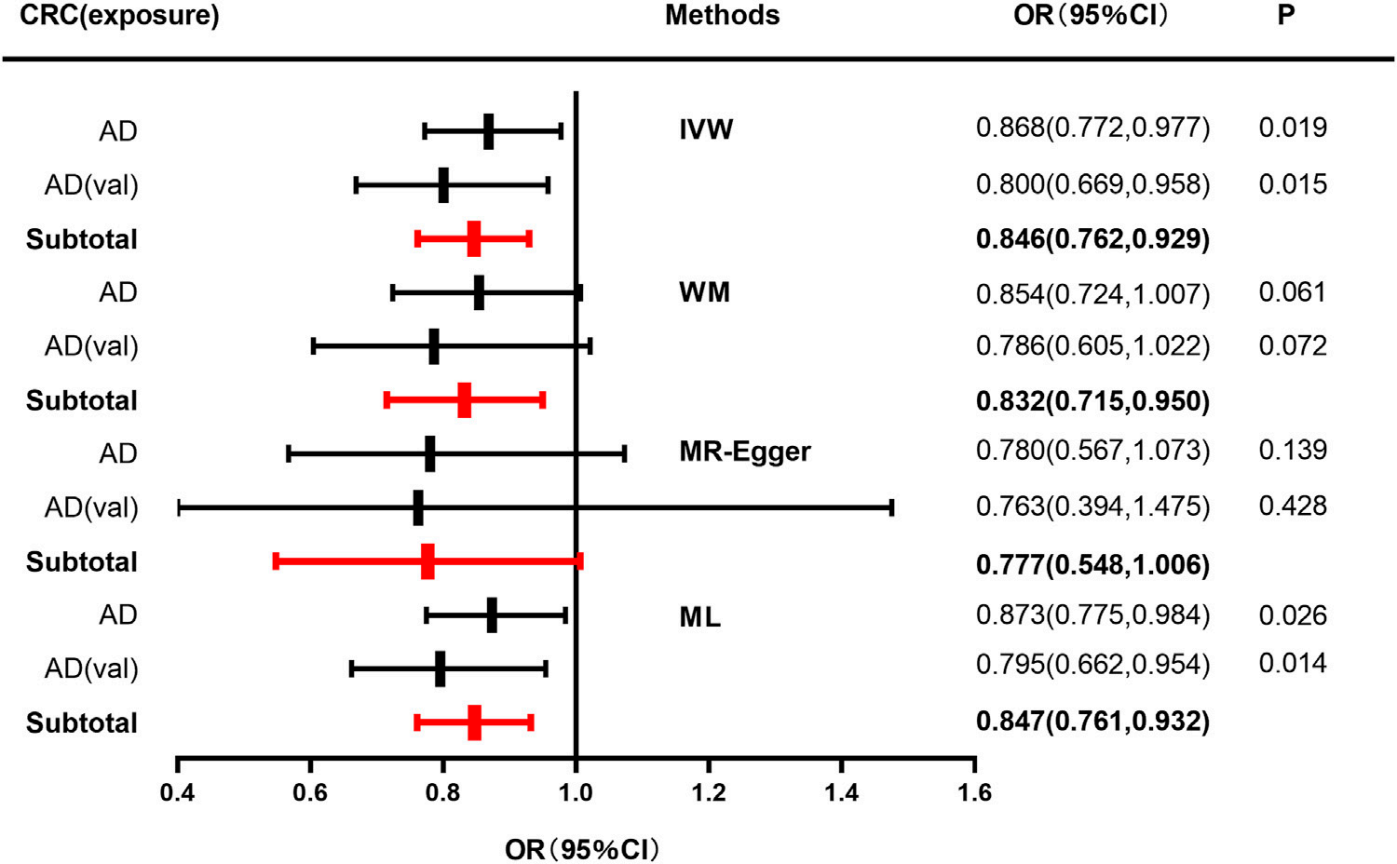
Forest plot to visualize causal effect of colorectal cancer on the risk of Alzheimer’s disease. For AD, a discovery sample (AD) and a validation sample (AD [val]) were used and the combined effect value was showed. Estimates are presented as odds ratios (ORs) and 95% CIs from four Mendelian randomization analyses methods including inverse-variance weighted (IVW), Weighted median (WM), Mendelian randomization egger (MR-Egger), Maximum likelihood (ML) and their subtotal results.) (Abbreviations: CRC, colorectal cancer; AD, Alzheimer’s disease; val, validation; OR, odds ratio; CI, confidence interval; P, *p*-value; IVW, inverse-variance weighted; MR-Egger, Mendelian randomization egger; ML, Maximum likelihood.).

**FIGURE 2:**
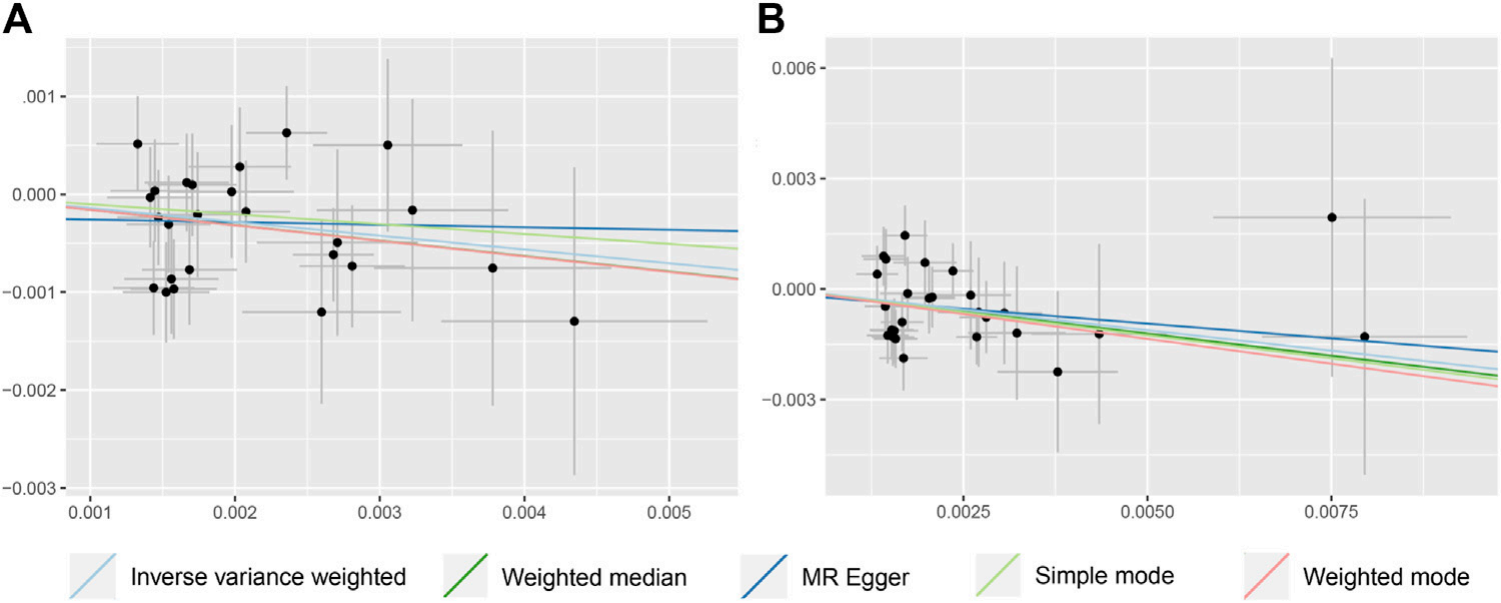
Scatter plot to visualize causal effect of colorectal cancer on the risk of Alzheimer’s disease **(A, B)**. Figures A and B respectively represent the causal effect of CRC on AD and AD (val), both estimated using five different statistical methods. The slope of the straight line indicates the magnitude of the causal association.

IVW, WM, MR-Egger, and ML methods were used to estimate the causal relationship between colorectal cancer (CRC) and Alzheimer’s disease (AD). In terms of the impact of CRC on AD, the two were significantly negatively correlated. The combined effect value of IVW showed that an increase of 1 log-odds in CRC led to a 15.4% reduction in the risk of AD (OR = 0.846, 95% CI: 0.762–0.929). Similar results were obtained with WM, MR-Egger regression, and ML methods, although some individual boundary values exceeded 1, they did not affect the overall results (Table 3; Figure 1). The forest plot was used to visualize the causal effect of each single SNP on the risk of Alzheimer’s disease, and the overall results were consistent with the previous findings (Supplementary Figures S1A, B). The slope of the regression line in the scatter plot also indicated a consistent causal effect direction, that is, CRC would reduce the risk of AD (Figures 2A, B).

**TABLE 3:** Mendelian randomization estimates for the effect of colorectal cancer on Alzheimer’s disease.

Outcome	Exposure	Method	OR	95%CI	*p*-value
AD	CRC	IVW	0.868	0.772–0.977	0.019
		Weighted median	0.854	0.724–1.007	0.061
		MR-Egger	0.780	0.567–1.073	0.139
		Maximum likelihood	0.873	0.775–0.984	0.026
AD (val)	CRC	IVW	0.800	0.669–0.958	0.015
		Weighted median	0.786	0.605–1.022	0.072
		MR-Egger	0.763	0.394–1.475	0.428
		Maximum likelihood	0.795	0.662–0.954	0.014

Abbreviations: CRC, colorectal cancer; AD, Alzheimer’s disease; val, validation; OR, odds ratio; CI, confidence interval; IVW, inverse-variance weighted; MR, mendelian randomization.

#### 3.2.2 Causal effect of AD on CRC

**FIGURE 3:**
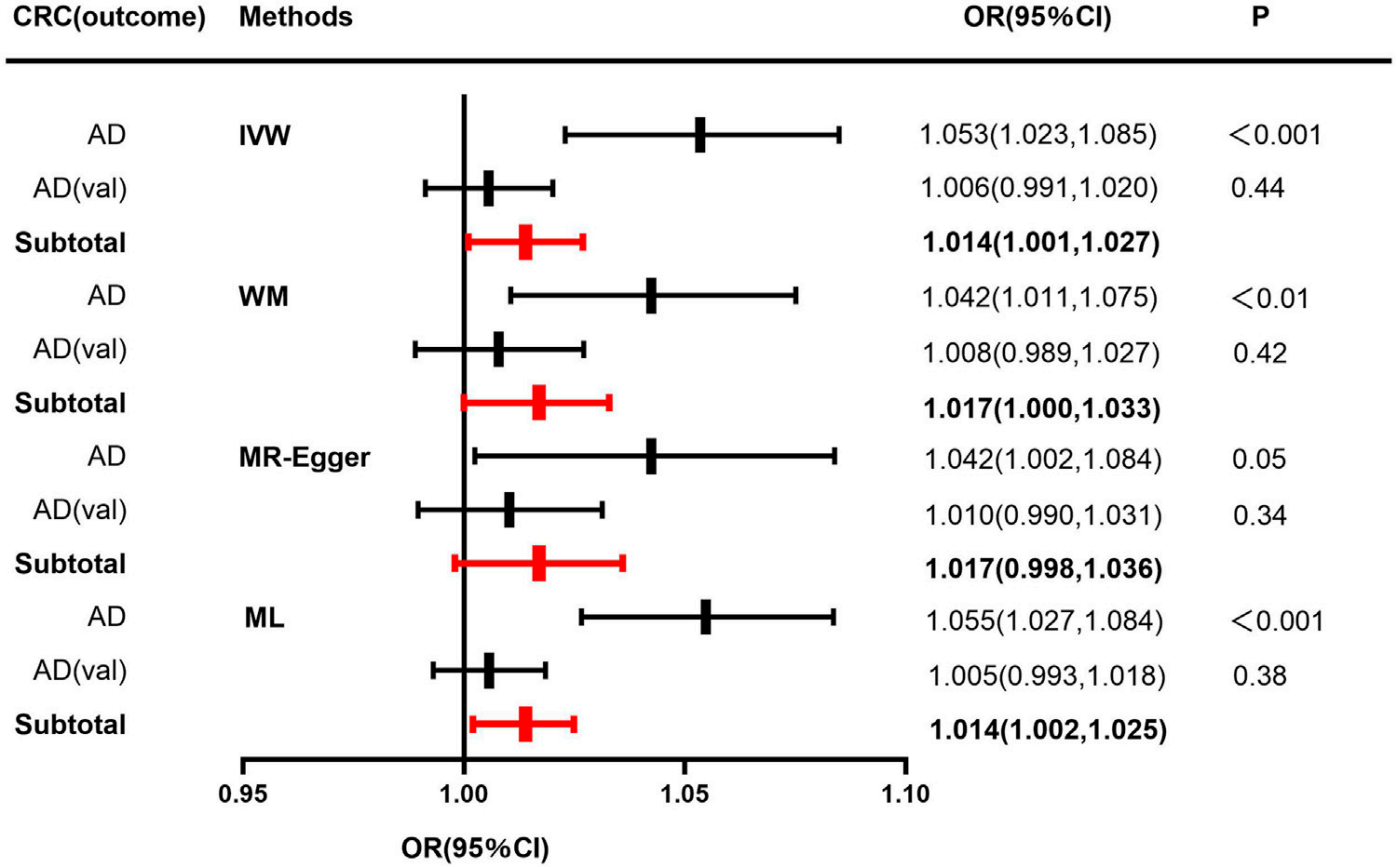
Forest plot to visualize causal effect of Alzheimer’s disease on the risk of colorectal cancer. For AD, a discovery sample (AD) and a validation sample (AD (val)) were used and the combined effect value was showed. Estimates are presented as odds ratios (ORs) and 95% CIs from four Mendelian randomization analyses methods including inverse-variance weighted (IVW), Weighted median (WM), Mendelian randomization egger (MR-Egger), Maximum likelihood (ML) and their subtotal results.) (Abbreviations: CRC, colorectal cancer; AD, Alzheimer’s disease; val, validation; OR, odds ratio; CI, confidence interval; P, *p*-value; IVW, inverse-variance weighted; MR-Egger, Mendelian randomization egger; ML, Maximum likelihood.).

**FIGURE 4:**
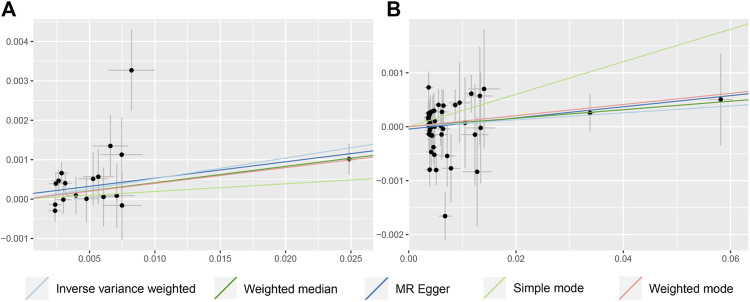
Scatter plot to visualize causal effect of colorectal cancer on the risk of Alzheimer’s disease **(C, D)**. Figures C and D respectively represent the causal effect of AD and AD (val) on CRC, both estimated using five different statistical methods. The slope of the straight line indicates the magnitude of the causal association.

In terms of the impact of AD on CRC, the two were positively correlated, although the effect size was small. The combined effect value of IVW showed that an increase of 1 log-odds in AD led to a 1.4% increase in the risk of CRC (OR = 1.014, 95% CI: 1.001–1.027). Similar results were obtained with WM, MR-Egger regression, and ML methods. However, in the AD (val) subgroup, multiple statistical methods showed no significant differences (Table 4; Figure 3), which was supported by the forest plot of single SNP evaluation (Supplementary Figures S1C, D). However, the slopes of regression lines for all methods in scatter plots indicated an increased risk of CRC in both subgroups AD and AD (val) (Figures 4C, D).

**TABLE 4:** Mendelian randomization estimates for the effect of Alzheimer’s disease on colorectal cancer.

Outcome	Exposure	Method	OR	95%CI	*p*-value
CRC	AD	IVW	1.053	1.023–1.085	<0.001
		Weighted median	1.042	1.011–1.075	0.009
		MR-Egger	1.042	1.002–1.084	0.054
		Maximum likelihood	1.055	1.027–1.084	<0.001
CRC	AD (val)	IVW	1.006	0.991–1.020	0.443
		Weighted median	1.008	0.989–1.027	0.415
		MR-Egger	1.010	0.990–1.031	0.338
		Maximum likelihood	1.005	0.993–1.018	0.379

Abbreviations: CRC, colorectal cancer; AD, Alzheimer’s disease; val, validation; OR, odds ratio; CI, confidence interval; IVW, inverse-variance weighted; MR-Egger, Mendelian randomization egger.

### 3.3 Sensitivity analyses

**FIGURE 5:**
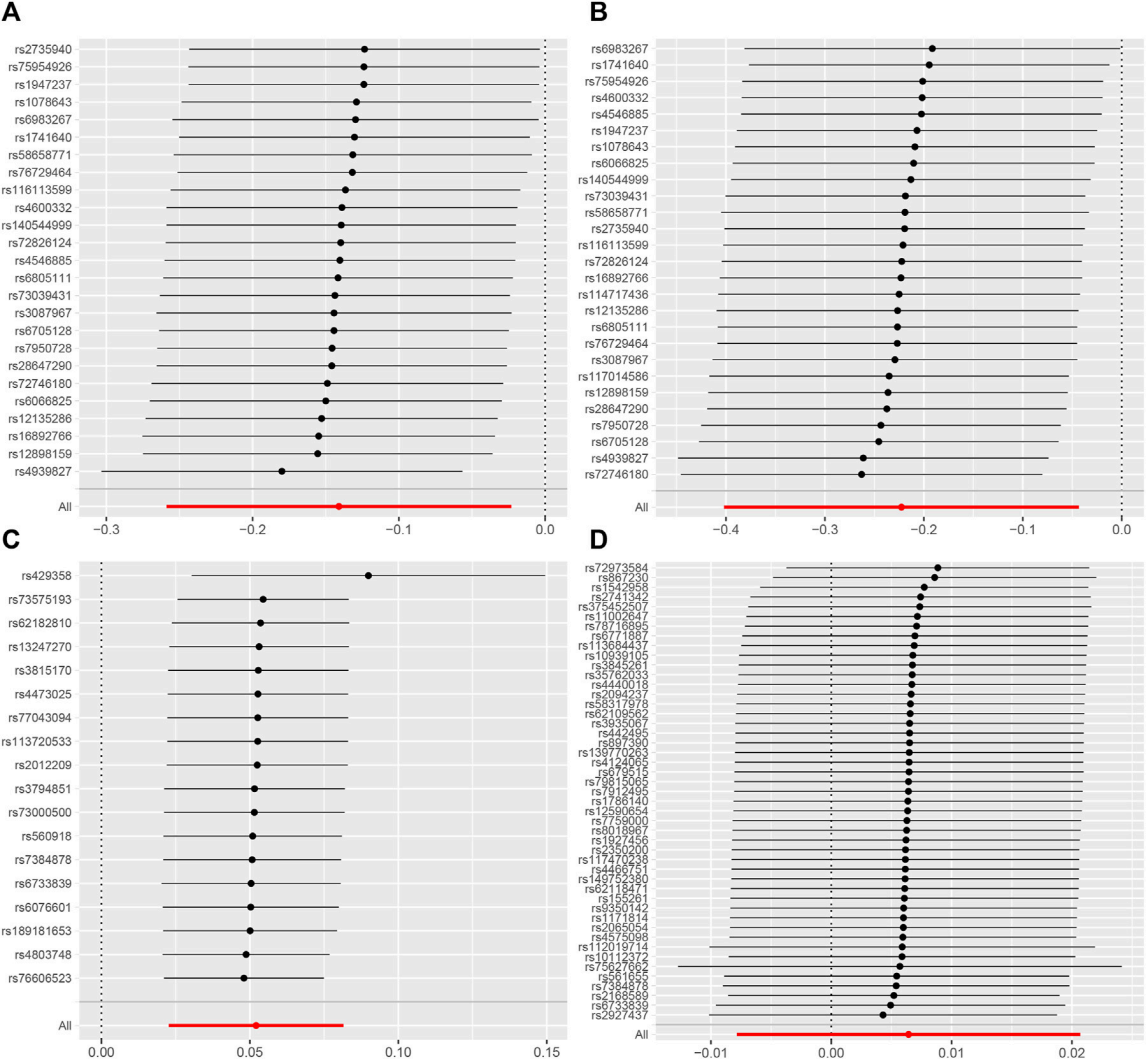
Leave-one-out plot to visualize causal effect of colorectal cancer on the risk of Alzheimer’s disease [**(A)** for AD subgroup and **(B)** for AD (val) subgroup] and *vice versa* (reverse direction) [**(C)** for AD subgroup and **(D)** for AD (val) subgroup] when leaving one SNP out.

The results from MRlap indicate that the MR results of AD on CRC are influenced by sample overlap, introducing bias and rendering these findings unreliable (Table 5). Cochran’s Q test of IVW and MR-Egger regression indicates no heterogeneity among SNPs (Table 6). The funnel plot shows a symmetrical distribution of points representing causal association effects when a single SNP is used as IV, suggesting a small likelihood of potential bias (Supplementary Figure S2). Sensitivity analyses using the “leave-one-out” approach reveals that the estimates of the remaining SNPs after excluding each SNP in turn are similar to those obtained with all SNPs included, indicating no SNPs with significant influence on the estimate of causal association (Figure 5). Therefore, all estimates of causal associations are reliable.

**TABLE 5:** Results of Sample Overlap Detection using MRlap.

Exposure	CRC	CRC	AD	AD (val)
Outcome	AD	AD (val)	CRC	CRC
OE	−0.047	−0.010	0.076	0.023
OEP	0.305	0.834	0.005	0.457
CE	−0.338	−0.202	−0.028	0.009
CEP	2.5e-05	0.009	0.455	0.807

Abbreviations: OE, observed effect; OEP, observed effect *p*-value; CE, corrected effect; CEP, corrected effect *p*-value.

**TABLE 6:** Heterogeneity of MR-Egger and IVW test.

Exposure	Outcome	Method	Q	df	*p*-value
CRC	AD	MR-Egger	20.11	23	0.64
CRC	AD	IVW	20.42	24	0.67
CRC	AD (val)	MR-Egger	24.76	25	0.48
CRC	AD (val)	IVW	24.82	26	0.53
AD	CRC	MR-Egger	19.72	16	0.23
AD	CRC	IVW	20.54	17	0.25
AD (val)	CRC	MR-Egger	59.54	45	0.07
AD (val)	CRC	IVW	60.03	46	0.08

Abbreviations: CRC, colorectal cancer; AD, Alzheimer’s disease; val, validation; MR-Egger, Mendelian randomization egger; IVW, inverse-variance weighted; df, degree of freedom; Q, heterogeneity statistic Q.

## 4 Discussion

This study represents the first MR study to evaluate the causal relationship between CRC and AD from a genetic susceptibility perspective. As mentioned in the research design, MR analyses require meeting three key assumptions: relevance, exclusion restriction, and independence. Therefore, statistical analyses revolve around these three core assumptions. Instruments selection and weak instrument testing have ensured relevance. Using the PhenoScanner website to exclude phenotypes, performing the intercept test in the MR-Egger regression model, and employing the MRPRESSO method to remove outliers all help to eliminate genetic pleiotropy, thus ensuring the exclusion restriction assumption. In terms of independence, testing the hypothesis rigorously can be challenging due to the lack of individual-level data and the inability to account for all confounding factors. Heterogeneity testing, leave-one-out analysis, and similar methods can be helpful for validation. One of the major concerns of MR is horizontal pleiotropy because the presence of genetic pleiotropy can introduce severe bias in MR analyses results (Verbanck et al., 2018; Rao et al., 2020). Therefore, in this study, we first used methods such as the PhenoScanner website, MR-Egger, and MRPRESSO to preliminarily exclude the pleiotropy of genes before conducting further MR analyses and sensitivity analyses. Our research findings indicate that genetic variations associated with CRC may potentially reduce the risk of AD. We also found evidence of an increased risk of CRC associated with AD, but the effect size was small and subgroup analyses were not statistically significant. Most importantly, the results of the MRlap analysis indicate a significant impact of sample overlap on the MR results, introducing substantial bias and rendering the results unreliable. Therefore, further research is needed to clarify the impact of AD on CRC. These results were supported by a series of sensitivity analyses exploring genetic pleiotropy, heterogeneity, and susceptibility. In general, current MR studies suggest that there may be a causal relationship between CRC and AD, and whether it is bidirectional still requires further investigation.

The incidence rate of CRC continues to increase with advancing age. The number of cases within each age group follows a bell-shaped distribution, with a peak occurring in the 60–74 age group (GBD, 2019 Colorectal Cancer Collaborators, 2022). Similarly, the incidence rate of AD also continues to rise with increasing age, with the ages of 60 or 65 and above being the high-risk age groups. These age ranges are also the focal points of AD research in various countries (Tahami Monfared et al., 2022). Both of them are common age-related diseases, with their incidence gradually increasing in old age. Interestingly, these two diseases, one representing ‘immortality’ of cells and the other representing ‘stagnation and death’ of cells, ultimately lead to the demise of the organism, as if it were an inevitable outcome that the human body cannot escape. Therefore, research on the correlation between these two diseases is of significant importance. There have been numerous observational studies focusing on the relationship between cancer and Alzheimer’s disease or central nervous system disorders. Most studies have shown a negative correlation between the two. However, there has been little research specifically addressing the link between colorectal cancer and Alzheimer’s disease (Driver et al., 2012; Catalá-López et al., 2014; Catalá-López et al., 2017; Ospina-Romero et al., 2020). Therefore, the present study holds significant importance. A meta-analysis indicated that individuals diagnosed with AD have a 42% reduced risk of developing cancer (95% CI, 0.40–0.86; *p* < 0.05), while patients with a history of cancer have a 37% reduced risk of developing AD (RR = 0.63; 95% CI, 0.56–0.72; *p* = 0.495), and the data did not show significant bias (Ma et al., 2014). In addition, a retrospective study discovered that individuals diagnosed with Alzheimer’s disease (AD) experience a decreased age-sex standardized rate of cancer development, which is not observed in individuals with Huntington’s disease (Panegyres and Chen, 2021). Furthermore, according to a cohort study involving more than 10,000 participants, individuals with AD had a 50% reduced risk of cancer, whereas cancer patients had a 35% lower risk of developing AD (Musicco et al., 2013). A MR study investigating the association between AD and gastrointestinal diseases revealed genetic correlations between cognitive traits and various gastrointestinal disorders (Adewuyi et al., 2022). All these pieces of evidence suggest a close and primarily negative correlation between CRC and AD; however, there is also evidence indicating that the negative correlation between the two may be influenced by real-world factors. Firstly, research suggests that the lower risk of AD among cancer patients may be attributed to underdiagnosis of AD (Freedman et al., 2016). On the other hand, many AD patients, especially male patients, cannot undergo colonoscopy in a timely manner due to cognitive dysfunction, memory loss, and emotional issues, and may not be diagnosed until the disease is advanced or even until death (Yang et al., 2021; Lv et al., 2022; Morishima et al., 2023). Secondly, research has found that chemotherapy for cancer survivors can reduce the risk of AD, such as the multi-target kinase inhibitor regorafenib used to treat CRC that can inhibit the inflammatory response caused by microglia and treat AD (Han et al., 2020; Akushevich et al., 2021). And some other drugs such as aspirin, metformin, angiotensin-converting enzyme inhibitors, and melatonin have benefits for both CRC and AD (Hybiak et al., 2020; Shafabakhsh et al., 2020; Naseri et al., 2022; Bueno and Frasca, 2023). Therefore, AD or CRC patients may be suppressing the occurrence of one disease while taking these drugs. Furthermore, research findings have indicated a significant positive correlation between the two. For example, a study found that CRC patients with vascular-related diseases may promote the occurrence of AD, and from 2000 to 2016, the number of patients with CRC who died from AD increased by 180 times in the United States (Lu et al., 2021; Du et al., 2022). Additionally, CRC patients with prolonged anesthesia exposure during abdominal or pelvic surgery have been reported to be associated with an elevated risk of developing AD (Akushevich et al., 2022). Another study has found that the incidence of various cancers increases rather than decreases in individuals at high risk for AD (Valentine et al., 2022). These studies are not consistent with the conclusion that they can reduce the risk of AD in CRC patients, but support the finding that AD patients, as indicated by the MR analyses in this study, may contribute to an increased risk of CRC. However, as previously mentioned, a subgroup result of the MR analysis for the latter is not statistically significant, so the relationship between the two requires further clinical and foundational research.

There is still controversy over whether CRC and AD have common biological mechanisms, but there are already some biological theories explaining the inverse correlation between cancer and AD (Ibáñez et al., 2014; Guo et al., 2017; Nudelman et al., 2019; Lanni et al., 2021). It has been suggested that PIN1 may serve as a unique and critical regulator connecting cancer and AD. studies on brain samples from individuals with mild cognitive impairment and AD have demonstrated significantly reduced levels of PIN1 expression, while PIN1 is typically overexpressed in various human cancers, such as colorectal cancer (Lu et al., 1999; Bao et al., 2004). Moreover, the Wnt signaling pathway and TMEFF2 methylation exhibit opposite activation states or effects in CRC and AD, while other factors such as Psen1, microRNA, methylation, mitochondrial oxidative stress, and blood-brain barrier ATP-binding cassette transporter proteins also play a regulatory role in both diseases (Tremolizzo et al., 2006; Thinnes, 2012; Aliev et al., 2013; Manandhar et al., 2020; Masood et al., 2020; Ghafouri-Fard et al., 2021; Kadkhoda et al., 2022). In recent years, research on the gut-brain axis has linked gastrointestinal diseases to AD. The gut microbiome dysbiosis leads to the secretion of amyloid proteins and lipopolysaccharides (LPS). This disrupts gut permeability and the blood-brain barrier, promotes neuroinflammation, and ultimately results in neuronal death in AD through inflammatory signaling pathways and neuronal damage (Kesika et al., 2021; Kim et al., 2021; Escobar et al., 2022; Thu Thuy Nguyen and Endres, 2022). However, few studies have explored the relationship between CRC gut microbiome dysbiosis and AD, which may be a critical point in the mechanisms underlying these two diseases.

In general, multiple clinical and basic research studies have demonstrated a close relationship between CRC and AD, with the main association being inverse. However, there are many biases in the real world, such as those related to economics, drugs, cognition, lifespan, and diagnosis, which have led some studies to question this relationship. Both CRC and AD are important diseases that lead to death in the elderly. Therefore, understanding their common mechanisms is of great significance for the treatment of these two diseases as well as longevity-related research.

## 5 Strengths and limitations

This study provides evidence for a causal relationship between CRC and AD in European ancestry populations. The use of publicly available GWAS data saves research costs and time and does not violate ethical principles. MR simulates the random allocation process and explores the causal relationship between the two diseases from a genetic etiology perspective, reducing bias caused by various confounding factors and reverse causality. However, this study has some limitations. Firstly, MR cannot further investigate and explain the biological mechanisms by which genetic variations affect the two diseases. Secondly, due to the lack of detailed individual data, subgroup analysis by age or gender cannot be performed. Furthermore, this study only includes populations of European ancestry, and therefore may not represent other populations such as those of Asian or African ancestry well.

## 6 Conclusion

In summary, our MR analyses revealed a decreased risk of Alzheimer’s disease (AD) in colorectal cancer (CRC) patients, while a slightly elevated risk of CRC in AD patients was observed. But due to the impact of sample overlap, the results of the latter are not reliable. Despite various biases inherent in real-world data, our results provide additional support for the opposing mechanisms of CRC and AD. Nonetheless, given the close relationship between these two diseases, our study highlights the importance of early diagnosis and treatment for both patient groups. Further investigations are warranted to elucidate the underlying pathophysiological mechanisms and treatment strategies for both diseases.

## Data Availability

The datasets presented in this study can be found in online repositories at IEU OpenGWAS project (https://gwas.mrcieu.ac.uk/) or GWAS Catalog (https://www.ebi.ac.uk/gwas/).
