# Supplementary figures and images for: Causal association between colorectal cancer and Alzheimer’s disease: a bidirectional two-sample mendelian randomization study
Source: Front Genet. 2024 Jan 5;14:1180905. doi: 10.3389/fgene.2023.1180905 (PMC10797121; doi:10.3389/fgene.2023.1180905)

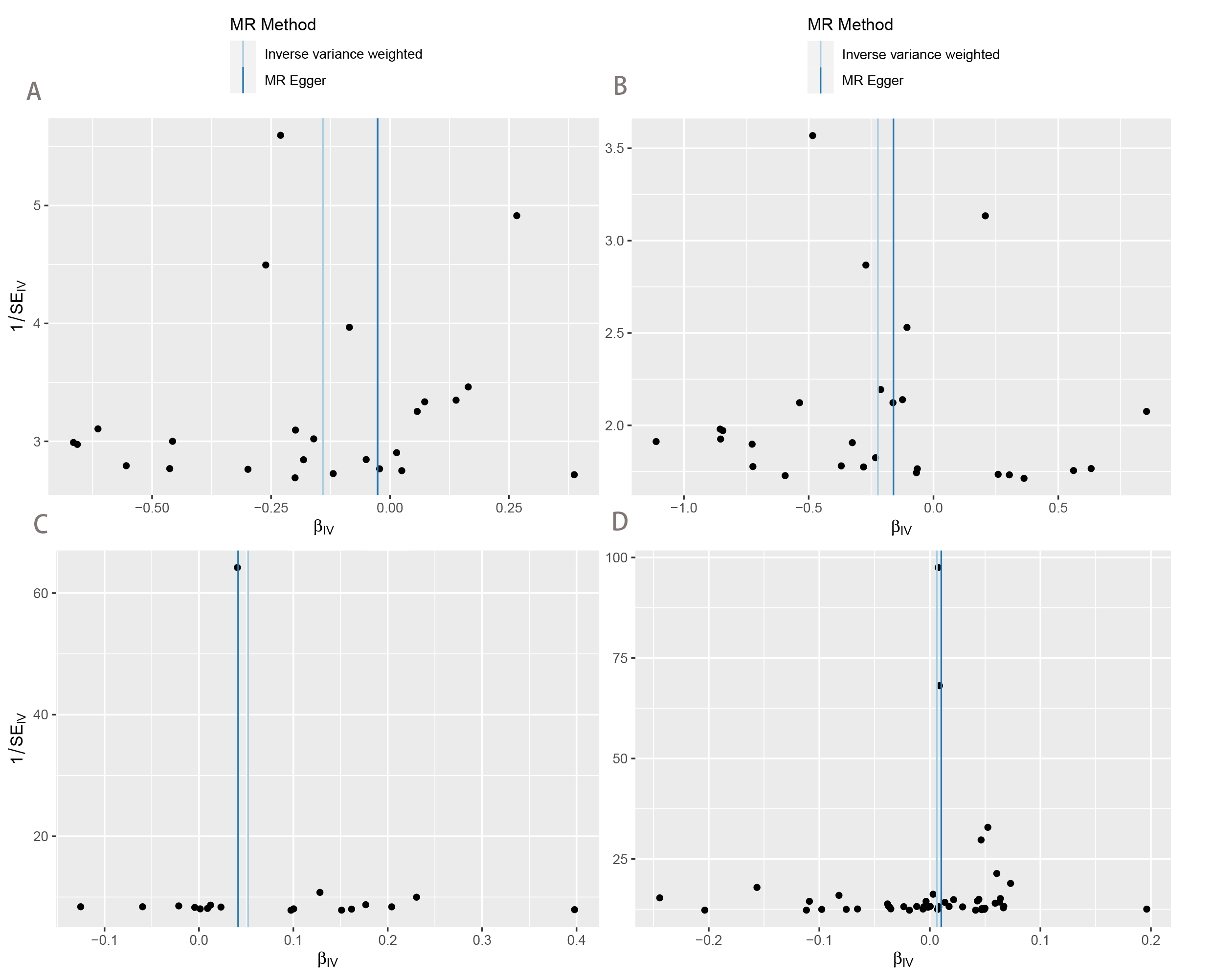

Supplement: Supplementary file 1 [file Image2.TIF]

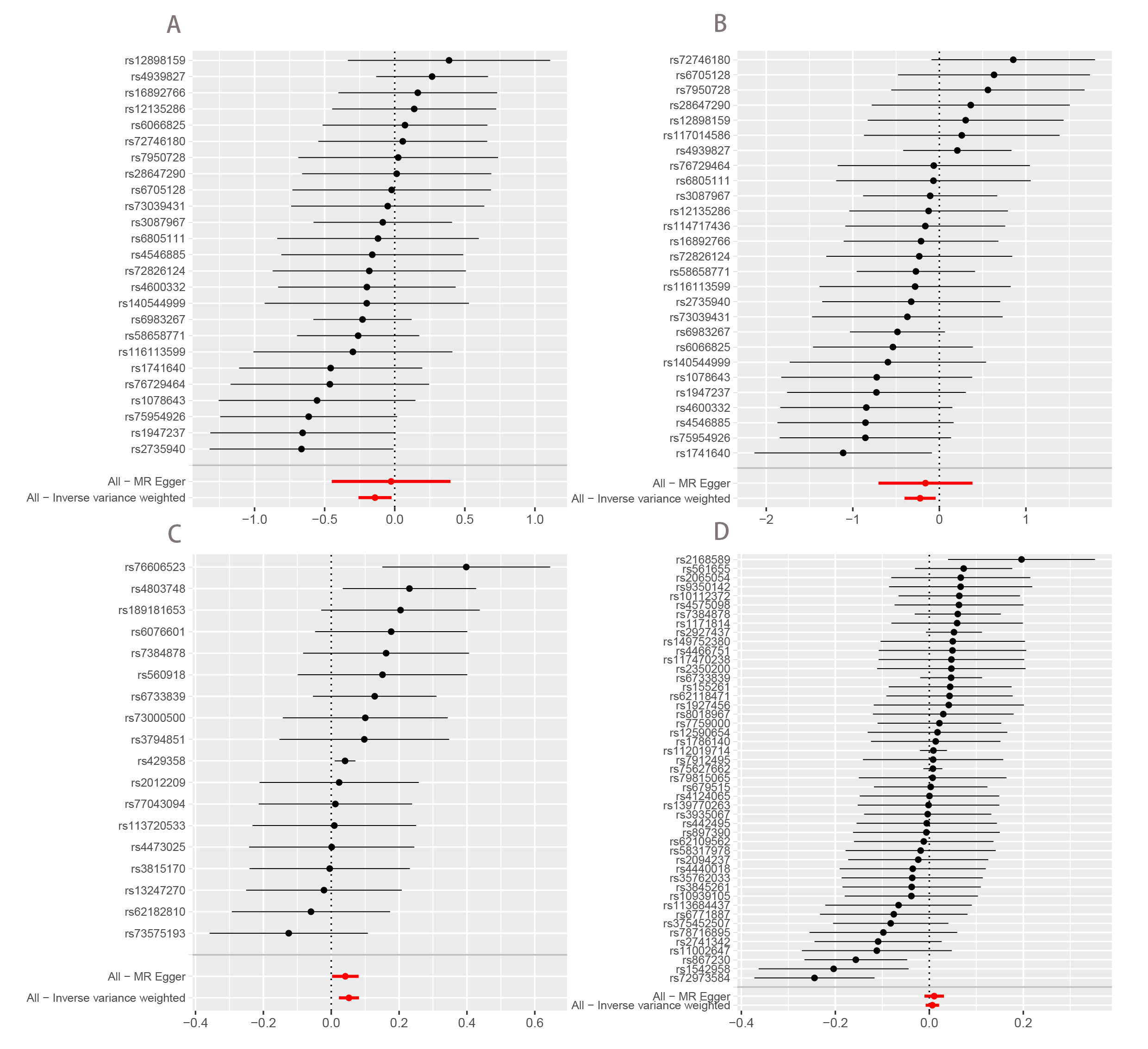

Supplement: Supplementary file 2 [file Image1.TIF]
