# Supplementary material for: Causal association between colorectal cancer and Alzheimer’s disease: a bidirectional two-sample mendelian randomization study
Source: Front Genet. 2024 Jan 5;14:1180905. doi: 10.3389/fgene.2023.1180905 (PMC10797121; doi:10.3389/fgene.2023.1180905)
Supplement: Supplementary file 3 [file DataSheet1.docx]

Supplementary Material

# Supplementary Figures and Tables

## Supplementary Figures

**Supplementary Figure 1.** Forest plot to visualize causal effect of each single SNP on the risk of Alzheimer’s disease (A for AD subgroup and B for AD (val) subgroup) and colorectal cancer (C for AD subgroup and D for AD (val) subgroup).

**Supplementary Figure 2.** Funnel plots to visualize overall heterogeneity of Mendelian randomization (MR) estimates for the effect of colorectal cancer on the risk of Alzheimer’s disease (A for AD subgroup and B for AD (val) subgroup) and vice versa (reverse direction) (C for AD subgroup and D for AD (val) subgroup).

## Supplementary Tables

**Supplementary Table 1** Association between the SNPs and colorectal cancer (outcome is Alzheimer's disease).

|  | SNP | EA | OA | pos | beta | se | *p*-Value |
| --- | --- | --- | --- | --- | --- | --- | --- |
| 1 | rs1078643 | A | G | 10707241 | 0.00156048 | 0.000325848 | 1.70E-06 |
| 2 | rs116113599 | A | G | 20722889 | -0.00434449 | 0.000919804 | 2.30E-06 |
| 3 | rs12135286 | T | C | 222218761 | 0.00203311 | 0.000354166 | 9.40E-09 |
| 4 | rs12898159 | T | C | 54415291 | 0.00132756 | 0.000285242 | 3.30E-06 |
| 5 | rs140544999 | A | G | 511139 | 0.00378033 | 0.000819956 | 4.00E-06 |
| 6 | rs16892766 | C | A | 117630683 | 0.00305526 | 0.000516081 | 3.20E-09 |
| 7 | rs1741640 | C | T | 60932414 | 0.0016853 | 0.000327831 | 2.70E-07 |
| 8 | rs1947237 | C | T | 142879468 | 0.00152417 | 0.000299172 | 3.50E-07 |
| 9 | rs2735940 | G | A | 1296486 | 0.00143794 | 0.000280776 | 3.00E-07 |
| 10 | rs28647290 | A | G | 67405582 | -0.00197669 | 0.000430577 | 4.40E-06 |
| 11 | rs3087967 | C | T | 111156836 | -0.00207534 | 0.000305631 | 1.10E-11 |
| 12 | rs4546885 | C | G | 183025555 | -0.00147091 | 0.000284338 | 2.30E-07 |
| 13 | rs4600332 | G | A | 111078872 | 0.00154139 | 0.000290444 | 1.10E-07 |
| 14 | rs4939827 | C | T | 46453463 | -0.00235666 | 0.000279728 | 3.60E-17 |
| 15 | rs58658771 | A | T | 33001734 | 0.00280944 | 0.000364099 | 1.20E-14 |
| 16 | rs6066825 | G | A | 47340117 | -0.00166639 | 0.000291721 | 1.10E-08 |
| 17 | rs6705128 | A | T | 199848803 | 0.00141443 | 0.000298341 | 2.10E-06 |
| 18 | rs6805111 | A | G | 67095678 | 0.00174157 | 0.000372906 | 3.00E-06 |
| 19 | rs6983267 | T | G | 128413305 | -0.0026797 | 0.00027963 | 9.40E-22 |
| 20 | rs7199157 | G | C | 155966 | -0.00129086 | 0.000280634 | 4.20E-06 |
| 21 | rs72746180 | C | A | 40240420 | 0.00170489 | 0.000305954 | 2.50E-08 |
| 22 | rs72826124 | A | G | 104916571 | 0.00270728 | 0.000556337 | 1.10E-06 |
| 23 | rs73039431 | G | A | 33523197 | -0.00322604 | 0.000661668 | 1.10E-06 |
| 24 | rs75954926 | G | A | 81061048 | 0.0015774 | 0.000296646 | 1.10E-07 |
| 25 | rs76729464 | A | G | 164959236 | 0.00259808 | 0.000548336 | 2.20E-06 |
| 26 | rs7950728 | C | T | 126644477 | -0.00144612 | 0.000306617 | 2.40E-06 |
| Abbreviations: SNP, Single Nucleotide Polymorphisms; EA, effect allele; OA, other allele; pos, position. | | | | | | | |

**Supplementary Table 2** Association between the SNPs and colorectal cancer (outcome is Alzheimer's disease validation).

|  | SNP | EA | OA | pos | beta | se | *p*-Value |
| --- | --- | --- | --- | --- | --- | --- | --- |
| 1 | rs1078643 | A | G | 10707241 | 0.00156048 | 0.000325848 | 1.70E-06 |
| 2 | rs114717436 | G | A | 28450527 | 0.00795214 | 0.00139467 | 1.20E-08 |
| 3 | rs116113599 | A | G | 20722889 | -0.00434449 | 0.000919804 | 2.30E-06 |
| 4 | rs117014586 | T | C | 69998965 | 0.00750852 | 0.00161475 | 3.30E-06 |
| 5 | rs12135286 | T | C | 222218761 | 0.00203311 | 0.000354166 | 9.40E-09 |
| 6 | rs12898159 | T | C | 54415291 | 0.00132756 | 0.000285242 | 3.30E-06 |
| 7 | rs140544999 | A | G | 511139 | 0.00378033 | 0.000819956 | 4.00E-06 |
| 8 | rs16892766 | C | A | 117630683 | 0.00305526 | 0.000516081 | 3.20E-09 |
| 9 | rs1741640 | C | T | 60932414 | 0.0016853 | 0.000327831 | 2.70E-07 |
| 10 | rs1947237 | C | T | 142879468 | 0.00152417 | 0.000299172 | 3.50E-07 |
| 11 | rs2735940 | G | A | 1296486 | 0.00143794 | 0.000280776 | 3.00E-07 |
| 12 | rs28647290 | A | G | 67405582 | -0.00197669 | 0.000430577 | 4.40E-06 |
| 13 | rs3087967 | C | T | 111156836 | -0.00207534 | 0.000305631 | 1.10E-11 |
| 14 | rs4546885 | C | G | 183025555 | -0.00147091 | 0.000284338 | 2.30E-07 |
| 15 | rs4600332 | G | A | 111078872 | 0.00154139 | 0.000290444 | 1.10E-07 |
| 16 | rs4939827 | C | T | 46453463 | -0.00235666 | 0.000279728 | 3.60E-17 |
| 17 | rs58658771 | A | T | 33001734 | 0.00280944 | 0.000364099 | 1.20E-14 |
| 18 | rs6066825 | G | A | 47340117 | -0.00166639 | 0.000291721 | 1.10E-08 |
| 19 | rs6705128 | A | T | 199848803 | 0.00141443 | 0.000298341 | 2.10E-06 |
| 20 | rs6805111 | A | G | 67095678 | 0.00174157 | 0.000372906 | 3.00E-06 |
| 21 | rs6983267 | T | G | 128413305 | -0.0026797 | 0.00027963 | 9.40E-22 |
| 22 | rs7199157 | G | C | 155966 | -0.00129086 | 0.000280634 | 4.20E-06 |
| 23 | rs72746180 | C | A | 40240420 | 0.00170489 | 0.000305954 | 2.50E-08 |
| 24 | rs72826124 | A | G | 104916571 | 0.00270728 | 0.000556337 | 1.10E-06 |
| 25 | rs73039431 | G | A | 33523197 | -0.00322604 | 0.000661668 | 1.10E-06 |
| 26 | rs75954926 | G | A | 81061048 | 0.0015774 | 0.000296646 | 1.10E-07 |
| 27 | rs76729464 | A | G | 164959236 | 0.00259808 | 0.000548336 | 2.20E-06 |
| 28 | rs7950728 | C | T | 126644477 | -0.00144612 | 0.000306617 | 2.40E-06 |
| Abbreviations: SNP, Single Nucleotide Polymorphisms; EA, effect allele; OA, other allele; pos, position. | | | | | | | |

**Supplementary Table 3** Association between the SNPs and Alzheimer's disease.

|  | SNP | EA | OA | pos | beta | se | *p*-Value |
| --- | --- | --- | --- | --- | --- | --- | --- |
| 1 | rs11234554 | G | C | 85849094 | 0.00245635 | 0.000480658 | 3.20E-07 |
| 2 | rs113720533 | T | C | 126963071 | -0.00606866 | 0.00127953 | 2.10E-06 |
| 3 | rs13247270 | A | G | 131809176 | 0.00748173 | 0.00150188 | 6.30E-07 |
| 4 | rs189181653 | A | G | 40190334 | 0.0065953 | 0.0013431 | 9.10E-07 |
| 5 | rs2012209 | T | C | 34385766 | 0.00395542 | 0.000812923 | 1.10E-06 |
| 6 | rs3794851 | G | C | 74709351 | -0.00527636 | 0.00115127 | 4.60E-06 |
| 7 | rs3815170 | A | G | 1013148 | -0.00299512 | 0.00061911 | 1.30E-06 |
| 8 | rs429358 | C | T | 45411941 | 0.0248558 | 0.000663291 | 1.0E-200 |
| 9 | rs4473025 | A | G | 103964996 | 0.00476063 | 0.00101267 | 2.60E-06 |
| 10 | rs4803748 | T | C | 45247048 | -0.00286042 | 0.00049505 | 7.60E-09 |
| 11 | rs560918 | T | C | 48104295 | 0.0074582 | 0.00162594 | 4.50E-06 |
| 12 | rs6076601 | A | G | 4054322 | 0.0026353 | 0.000517113 | 3.50E-07 |
| 13 | rs62182810 | A | G | 204387482 | 0.00236839 | 0.000482703 | 9.30E-07 |
| 14 | rs6733839 | T | C | 127892810 | 0.00313752 | 0.000499313 | 3.30E-10 |
| 15 | rs73000500 | T | C | 58791675 | -0.00566797 | 0.0012046 | 2.50E-06 |
| 16 | rs73575193 | T | C | 70297568 | -0.00235674 | 0.000480557 | 9.40E-07 |
| 17 | rs7384878 | T | C | 99932049 | 0.0024243 | 0.000518463 | 2.90E-06 |
| 18 | rs76606523 | A | G | 77124806 | 0.00820885 | 0.00177585 | 3.80E-06 |
| 19 | rs77043094 | T | C | 186935749 | 0.00707863 | 0.00139886 | 4.20E-07 |
| Abbreviations: SNP, Single Nucleotide Polymorphisms; EA, effect allele; OA, other allele; pos, position. | | | | | | | |

**Supplementary Table 4** Association between the SNPs and Alzheimer's disease validation.

|  | SNP | EA | OA | pos | beta | se | *p*-Value |
| --- | --- | --- | --- | --- | --- | --- | --- |
| 1 | rs10112372 | T | A | 139351509 | -0.00447785 | 0.000795664 | 1.30E-08 |
| 2 | rs10939105 | G | A | 11023682 | -0.00437249 | 0.000850207 | 1.80E-07 |
| 3 | rs11002647 | T | C | 80491843 | 0.00418524 | 0.000913983 | 3.90E-06 |
| 4 | rs112019714 | C | T | 45404857 | 0.0581685 | 0.0022844 | 6.1E-143 |
| 5 | rs113684437 | G | C | 16744130 | -0.0127591 | 0.00271563 | 2.60E-06 |
| 6 | rs1171814 | T | G | 61645833 | -0.00394242 | 0.00075608 | 1.90E-07 |
| 7 | rs117470238 | A | G | 112983458 | -0.00948287 | 0.00201597 | 2.80E-06 |
| 8 | rs12590654 | A | G | 92938855 | -0.00391784 | 0.000798343 | 1.30E-06 |
| 9 | rs139770263 | T | C | 211543655 | 0.0134099 | 0.00275456 | 1.30E-06 |
| 10 | rs149752380 | A | T | 235458057 | 0.0140544 | 0.0029661 | 2.90E-06 |
| 11 | rs1542958 | A | C | 50823322 | -0.00391405 | 0.00085699 | 2.80E-06 |
| 12 | rs155261 | G | T | 1843069 | 0.00620158 | 0.0012931 | 2.20E-06 |
| 13 | rs1786140 | C | T | 59908627 | -0.00403296 | 0.000760903 | 6.70E-08 |
| 14 | rs1927456 | C | A | 30008609 | -0.00364448 | 0.000798129 | 4.80E-06 |
| 15 | rs2065054 | T | C | 53201631 | -0.00371784 | 0.000753832 | 8.90E-07 |
| 16 | rs2094237 | C | T | 107877569 | -0.00612484 | 0.00124297 | 6.40E-07 |
| 17 | rs2168589 | A | T | 50270105 | 0.00371767 | 0.000797108 | 1.80E-06 |
| 18 | rs2350200 | C | T | 5943465 | -0.00365549 | 0.000791414 | 3.10E-06 |
| 19 | rs2741342 | T | C | 27330096 | -0.00476554 | 0.000881833 | 1.10E-07 |
| 20 | rs2927437 | G | A | 45241638 | -0.0116763 | 0.000951526 | 1.30E-34 |
| 21 | rs35762033 | A | G | 19803080 | -0.00429294 | 0.000882935 | 1.40E-06 |
| 22 | rs375452507 | A | T | 31115259 | -0.00462042 | 0.00077905 | 4.50E-09 |
| 23 | rs3845261 | T | C | 5008236 | 0.00397542 | 0.000802136 | 2.20E-07 |
| 24 | rs3935067 | C | G | 143104331 | 0.00417107 | 0.000773346 | 5.80E-08 |
| 25 | rs4124065 | T | G | 6128400 | -0.00482386 | 0.000985519 | 8.40E-07 |
| 26 | rs442495 | C | T | 59022615 | -0.00393814 | 0.000806353 | 1.30E-06 |
| 27 | rs4440018 | G | A | 135437405 | -0.00377755 | 0.000810051 | 1.80E-06 |
| 28 | rs4466751 | T | C | 110289323 | 0.00376053 | 0.000809026 | 4.80E-06 |
| 29 | rs4575098 | A | G | 161155392 | 0.00471988 | 0.00088687 | 2.50E-07 |
| 30 | rs561655 | A | G | 85800279 | 0.00555837 | 0.000788858 | 1.40E-12 |
| 31 | rs58317978 | C | T | 28812268 | -0.00390734 | 0.000854881 | 4.30E-06 |
| 32 | rs62109562 | G | T | 45913737 | 0.0123535 | 0.00253729 | 1.20E-06 |
| 33 | rs62118471 | C | T | 45691049 | -0.0132637 | 0.00245042 | 6.70E-08 |
| 34 | rs6733839 | T | C | 127892810 | 0.0086699 | 0.000781608 | 4.60E-28 |
| 35 | rs6771887 | A | G | 135798396 | -0.00717704 | 0.00154061 | 2.60E-06 |
| 36 | rs679515 | C | T | 207750568 | -0.00594473 | 0.000983656 | 1.50E-09 |
| 37 | rs72973584 | T | C | 1046076 | 0.00678091 | 0.00118483 | 8.90E-09 |
| 38 | rs7384878 | T | C | 99932049 | 0.00647363 | 0.000811953 | 7.10E-16 |
| 39 | rs75627662 | T | C | 45413576 | 0.0337993 | 0.000927484 | 1.0E-200 |
| 40 | rs7759000 | C | T | 22291959 | 0.00486602 | 0.000877348 | 3.50E-08 |
| 41 | rs78716895 | T | A | 162866561 | 0.00787989 | 0.00170626 | 4.90E-06 |
| 42 | rs7912495 | G | A | 11718713 | 0.00370372 | 0.000758054 | 8.20E-07 |
| 43 | rs79815065 | A | G | 46384317 | 0.0104995 | 0.00226341 | 2.30E-06 |
| 44 | rs8018967 | C | T | 73976934 | 0.0038617 | 0.000792342 | 6.80E-07 |
| 45 | rs867230 | A | C | 27468503 | 0.00513562 | 0.000768974 | 3.70E-11 |
| 46 | rs897390 | C | T | 123329690 | 0.00640098 | 0.00137475 | 1.50E-06 |
| 47 | rs9350142 | G | A | 19435682 | 0.00405976 | 0.00084764 | 1.20E-06 |
| Abbreviations: SNP, Single Nucleotide Polymorphisms; EA, effect allele; OA, other allele; pos, position. | | | | | | | |
